# Prenatal diagnosis of sirenomelia in the first trimester: A case report

**DOI:** 10.4274/tjod.90688

**Published:** 2016-03-10

**Authors:** Yasin Ceylan, Yasemin Doğan, Sebiha Özkan Özdemir, Gülseren Yücesoy

**Affiliations:** 1 Kocaeli University Faculty of Medicine, Department of Obstetrics and Gynecology, Kocaeli, Turkey

**Keywords:** Sirenomelia, congenital syndrome, Prenatal diagnosis

## Abstract

Sirenomelia or “mermaid syndrome” is a rare congenital syndrome characterized by the anomalous development of the caudal region of the body. We present a case of sirenomelia diagnosed in the first trimester using two-dimensional and three-dimensional ultrasonographic examination. A nulliparous woman aged thirty years was referred to our perinatology unit for evaluation because of oligohydramnios at 12 weeks of gestation. Her medical history was unremarkable. There was no family history of genetic abnormalities. We identified a single lower extremity and severe oligohydramnios, which are characteristics of sirenomelia. Sirenomelia, a developmental defect involving the caudal region of the body, is associated with several internal visceral anomalies. Sirenomelia is fatal in most cases due to the characteristic pulmonary hypoplasia and renal agenesia. Prenatal diagnosis of sirenomelia may be difficult in the second or third trimester because of the severe oligohydramnios; it should be easier to diagnose sirenomelia in the first trimester.

## PRECIS:

A diagnosis of sirenomelia may be easier to make during the first trimester because the amniotic fluid volume is relatively normal. During later periods, ultrasonographic diagnosis may be prevented because of severe oligohydramnios due to renal agenesis or dysgenesis.

## INTRODUCTION

Sirenomelia or “mermaid syndrome” is a rare congenital syndrome characterized by the anomalous development of the caudal region of the body^(1)^. The syndrome has been reported to occur 1 in 60 000 live births, and predominantly in male fetuses^(2)^. Progressive oligohydramnios is usually the first sign of this syndrome in the second trimester because of renal abnormalities^(3)^. Sirenomelia should be easier to diagnose in the first trimester. We present a case of sirenomelia diagnosed in the first trimester using two-dimensional and three-dimensional ultrasonographic examination.

## CASE REPORT

**Figure 1:**
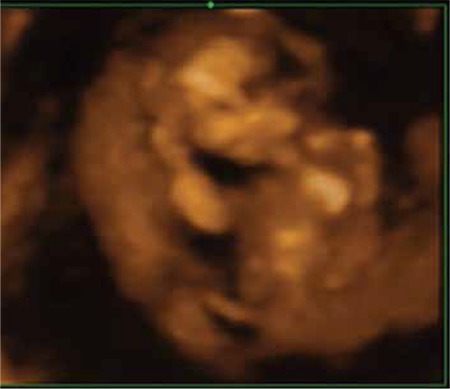
Three dimensional ultrasonographic image shows normal upper limbs and single lower limb

**Figure 2:**
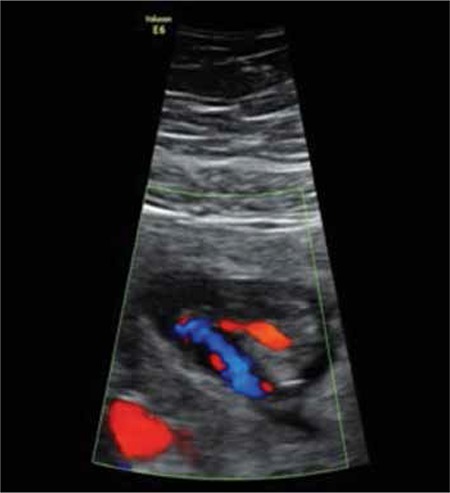
Color doppler ultrasonographic image shows single umblical artery

**Figure 3:**
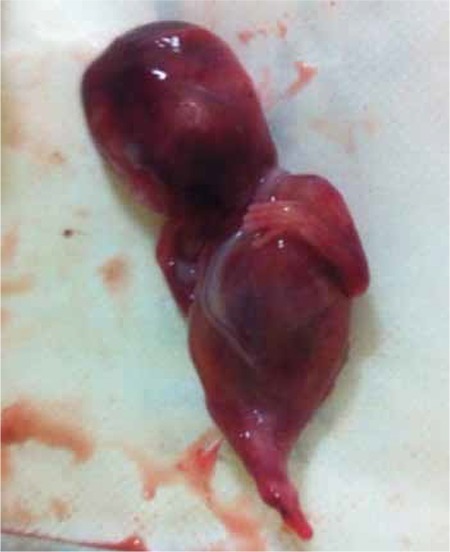
Examination of the fetus

A nulliparous woman aged 30 years was referred to our perinatology unit for evaluation because of oligohydramnios at 12 weeks of gestation. An antenatal ultrasonographic scan revealed a single live fetus with oligohydramnios. The upper half of the fetus appeared normal with both upper extremities seen separately and moving normally. However, the lower extremities appeared to be fused together in fixed extension and mobility at the hip and knee joints was restricted (Figure 1). A single umbilical artery was demonstrated using color Doppler (Figure 2). Thus, the diagnosis of sirenomelia was made prenatally. Medical termination of pregnancy was performed with the informed decision of the parents. On postnatal examination, the fetus weighed 150 grams. External examination revealed fusion of both lower extremities (Figure 3). Based on the external examination, sirenomelia was diagnosed.

## DISCUSSION

Sirenomelia, a developmental defect that involves the caudal region of the body, is associated with several internal visceral anomalies. It is associated with renal agenesis, sacral agenesis, anorectal atresia, imperforate anus, absent urinary bladder, lumbosacral and pelvic bone abnormalities, single umbilical artery, and ambiguous genitalia. Sirenomelia is fatal in most cases because of the characteristic pulmonary hypoplasia due to the severe oligohydramnios^(4)^. Two theories have been proposed to explain the etiopathogenesis of sirenomelia; the vascular steal hypothesis and the defective blastogenesis hypothesis. Normally, the umbilical cord contains two arteries that originate from the iliac arteries, which return blood to the placenta. In cases of sirenomelia, the umbilical artery is single and arises from the abdominal aorta. The abdominal aorta distal to this branch directly bifurcates into iliac iliac arteries without giving an origin to renal or inferior mesenteric artery branches. These vascular abnormalities lead to vitelline artery steal of the blood supply to the caudal end of embryo, which leads to sirenomelia and associated anomalies^(5)^. At blastogenesis, damage to the caudal mesoderm of the embryo between day 13 and day 22 of life results in merging, malrotation, and dysgenesis of the lower extremities.

A diagnosis of sirenomelia may be easier to make during the first trimester because the amniotic fluid volume is relatively normal, because amniotic fluid is secreted by the amniotic membrane in the first trimester^(6)^. A diagnosis of sirenomelia is made in early pregnancy through confirmation of the existence of a single lower extremity. At later periods of pregnancy, ultrasonographic diagnosis of sirenomelia may be prevented by severe oligohydramnios due to renal agenesis or dysgenesis. Our patient was referred to us late in the first trimester.
